# Continuous Glucose Monitoring vs. Capillary Blood Glucose in Hospitalized Type 2 Diabetes Patients

**DOI:** 10.7759/cureus.43832

**Published:** 2023-08-21

**Authors:** David Veríssimo, Joana Vinhais, Catarina Ivo, Ana Cláudia Martins, João Nunes e Silva, Dolores Passos, Luís Lopes, João Jácome de Castro, Mafalda Marcelino

**Affiliations:** 1 Department of Endocrinology, Hospital das Forças Armadas, Lisbon, PRT

**Keywords:** time-in-range, inpatient diabetes management, glycemic control, continuous glucose monitoring, capillary blood glucose

## Abstract

Introduction: The emergence of continuous glucose monitoring devices revolutionized the monitoring of diabetes, allowing real-time measurement of interstitial glucose levels. These devices are especially important for people with diabetes treated with insulin therapy and have been extensively studied in outpatient settings. In hospitalized patients, studies using continuous glucose monitoring have focused mainly on evaluating its accuracy and feasibility, but the results were unclear on whether continuous glucose monitoring was superior to capillary blood glucose in improving glycemic control and further research is needed to support the use of these devices in hospitalized patients with diabetes.

Objective: The primary endpoint of this study was to assess the increase in time-in-range (glycemic readings between 100-180 mg/dL) in hospitalized patients with continuous glucose monitoring, compared to capillary blood glucose. The secondary endpoints included the assessment of reductions in hypoglycemia incidence, mean glucose levels, and glucose coefficient of variation. Additionally, we assessed the intervention's impact on reducing the length of hospital stay, mortality rates, and incidence of inpatient infections.

Research design and methods: This was a retrospective, cohort study of 60 hospitalized patients with type 2 diabetes, divided into two groups of 30 individuals each: an intervention group monitored through continuous glucose monitoring and a control group using capillary blood glucose.

Results: Both groups were comparable in terms of demographic and clinical characteristics. Continuous glucose monitoring users had a higher number of readings per day (six vs. four, *p *< 0.001), in-range readings (53.5% vs. 35%, *p *= 0.027), fewer above-range readings (25.5% vs. 56.5%, *p *= 0.003), particularly above 250 mg/dL (5% vs. 27.5%, *p *= 0.001), with no difference in the percentage of hypoglycemia occurence (1% vs. 0%, *p *= 0.107). Lower mean glucose (161.9 mg/dL vs. 206.5 mg/dL, *p *< 0.001) was also observed in this group. No difference was observed in mortality, length of stay, or in infection rate (*p *= 1.000, *p *= 0.455, and *p *= 0.606, respectively).

Conclusions: This retrospective study supports the use of continuous glucose monitoring in optimizing glycemic control in hospitalized patients with type 2 diabetes on intensive insulin therapy. These findings suggest that continuous glucose monitoring can improve time-in-range and prevent hyperglycemia.

## Introduction

Type 2 diabetes is a chronic and progressive disease characterized by a state of hyperglycemia associated with micro and macrovascular complications. In clinical practice, the main objective is to ensure that patients maintain optimal glycemic control in order to prevent these complications [1]. Good control implies a constant optimization of therapy, including insulin therapy, when needed. However, this necessary intensification of insulin therapy is often associated with a decrease in quality of life and an increase in treatment costs, and the risk of hypoglycemia [2].

In hospitalized patients, in whom both hyperglycemia and hypoglycemia are associated with increased morbidity, mortality, and length of hospital stay [3], an insulin regimen is the preferred treatment [1]. However, this management can be challenging due to acute illnesses, as well as various procedures, medications, and dietary changes, including prolonged fasting periods [1].

Capillary blood glucose (CBG) is recommended by the American Diabetes Association in Standards of Care in Diabetes 2023 as a method for diabetes control in non-critically ill hospitalized patients with type 2 diabetes [1]. However, since CBG measures only immediate blood glucose levels, it has limitations because it may not provide sufficient information, including the detection of hypoglycemia (often asymptomatic), severe hyperglycemia, night patterns, and glycemic fluctuations. CBG is also an invasive and painful procedure, especially when performed several times a day [2], as normally recommended [1]. The emergence of continuous glucose monitoring (CGM) devices revolutionized the monitoring of diabetes, allowing real-time measurement of interstitial glucose levels [4].

These devices are especially important for people with diabetes treated with insulin therapy [5]. CGM has been extensively studied in outpatient settings and has shown improved glycemic control by reducing hypoglycemia and increasing time-in-range in both type 1 and type 2 diabetes [6-9].

In hospitalized patients, studies using CGM have focused mainly on evaluating its accuracy and feasibility, with promising results. A study by Davis et al., using real-time CGM (rtCGM), obtained accurate glucose measurements, with a mean absolute relative difference (MARD) of 12.8% between CGM and reference glucose values [10]. Similarly, Murray-Bachmann et al. observed a MARD of 13.2% with an intermittently scanned CGM (isCGM) device [11]. Despite the described outcomes, the results were unclear on whether CGM was superior to CBG in improving glycemic control, and further research was needed to support the use of CGM devices in hospitalized patients with diabetes.

To minimize the exposure of healthcare personnel during the coronavirus disease 2019 (COVID-19) pandemic, our hospital utilized isCGM technology to monitor glycemic control in patients with diabetes. At the end of this period and to address the aforementioned gap in the literature, we decided to conduct a cohort study aiming to compare the efficacy of isCGM devices versus CBG in glycemic control of inpatient type 2 diabetes on intensive insulin therapy.

## Materials and methods

Research design

This study aimed to evaluate the impact of FreeStyle Libre 2 (Abbott Laboratories Chicago, Illinois, United States), an isCGM device, compared to CBG on glycemic control in adults with type 2 diabetes exclusively on intensive insulin therapy, during hospitalization. The study was designed as a retrospective cohort study and data was obtained by analyzing the clinical records of hospitalized patients in the Hospital das Forças Armadas - Pólo de Lisboa, Lisbon, Portugal. We collected admission baseline patient data, including age, sex, admission diagnosis, as well as diabetes duration, control, complications, and treatment.

Patient selection

Eligible patients were randomly chosen among type 2 diabetes patients, 18 years or older, capable of oral feeding, and hospitalized for at least seven days in general medicine or surgery wards, between January and December 2021. They were divided into two groups: The first group had 30 patients who were being monitored using a Freestyle Libre 2 device and the second was a control group of 30 patients with the same characteristics, but monitored using CBG, randomly chosen from the same population.

Study objectives

The primary objective of this study was to evaluate the effect of glucose monitoring in time-in-range, defined as the percentage of glucose readings falling between 100 mg/dL and 180 mg/dL according to our hospital inpatient protocol. Secondary endpoints included reductions in hypoglycemic events (percentage of glucose readings below 70 mg/dL and 55 mg/dL), time spent above the target range (percentage of glucose readings above 180 mg/dL and 250 mg/dL), mean glucose levels, and glucose coefficient of variation. All CGM readings below 70 mg/dL and 55 mg/dL were confirmed by CBG. Additionally, we assessed the intervention's impact on reducing the length of hospital stay, mortality rates, and the incidence of inpatient infections. These outcomes were assessed to determine the overall efficacy of glucose monitoring in managing glycemic control.

Statistical analysis

Quantitative data were presented as means and standard deviation or medians and quartiles, according to their adaptation to a normal distribution, which was evaluated by the Shapiro-Wilk test. In accordance with this, comparisons of independent samples were performed using the Student's t-test after evaluation of homoscedasticity (Levene's test) or using the Mann-Whitney U test, while pairwise comparisons were performed applying the paired t-test or the Wilcoxon test. Qualitative data were presented as absolute and relative frequencies and associations between independent subgroups were analyzed using Fisher's exact test. The analysis was performed using the R language 4.2.1 in the RStudio environment (2022.07.0+548; RStudio, PBC, Boston, Massachusetts, United States) and was evaluated at a significance level of 5%.

## Results

Baseline characteristics (Table 1) were similar between the CGM and CBG groups, with no significant differences in age (74.4 vs 76.8 years, p = 0.277), sex (83.33% vs. 63.33% male, p = 0.143), admission diagnosis (p = 0.346), duration of type 2 diabetes (13.5 vs. 12 years, p = 0.824), nor type 2 diabetes complications (p = 0.151) as measured by the Diabetes Complications Severity Index [12]. All the patients were diagnosed with type 2 diabetes before hospitalization, and most patients in both groups were being treated with non-insulin antidiabetic medications (53.3% vs. 70%). During hospitalization, the main admission diagnosis was infectious disease in both groups (53.3% and 48.3%). The use of corticosteroids was similar between groups (p = 1.000).

**Table 1:** Baseline Characteristics of the Patients CGM: continuous glucose monitoring; CBG: capillary blood glucose; IQR: interquartile range; COVID-19: coronavirus disease 2019 *Student’s t test for independent samples; **Fisher exact test; ***Mann-Whitney U test

Characteristic			CGM (n = 30)	CBG (n = 30)	p-value
Age (years), mean ± SD			76.8 ± 6.9	74.4 ± 9.4	0.277*
Male sex, n (%)			25 (83.3)	19 (63.3)	0.143**
Diabetes duration (years), median (IQR)			12 [2; 22.8]	13.5 [6.3;23]	0.824***
Diabetes ambulatory treatment, n (%)					
	Diet/Lifestyle measures		3 (10)	3 (10)	0.570**
	Non-insulin medications		21 (70)	16 (53.3)	
	Basal insulin		3 (10)	6 (20)	
	Intensive insulin		3 (10)	5 (16.7)	
Diabetes complications, n (%)					
	Retinopathy		2 (6.7)	6 (20)	0.254**
	Nephropathy		13 (43.3)	13(43.3)	1.000**
	Neuropathy		1 (3.3)	2 (6.7)	1.000**
	Cardiovascular disease		7 (23.3)	9 (30)	0.770**
	Cerebrovascular disease		5 (16.7)	7 (23.3)	0.748**
	Peripheral arterial disease		6 (20)	8 (26.7)	0.761**
	Metabolic complications		0 (0)	2 (6.7)	0.492**
Diabetes Complications Severity Index, median (IQR)			1 [0; 4]	2 [0; 5.3]	0.151***
Admission diagnosis, n (%)					
	Infectious		16 (53.3)	14 (48.3)	0.157**
		COVID-19	5 (16.7)	11 (36.7)	
	Cardiovascular		9 (30)	6 (20.7)	
	Gastrointestinal		3 (10)	2 (6.9)	
	Oncologic		1 (3.3)	7 (24.1)	
	Metabolic		1 (3.3)	0 (0)	
Admission laboratory results					
	Hemoglobin (g/dL), median (IQR)		12.6 (9.8; 13.9)	11.7 (9.5; 12.9)	0.174***
	Glucose (mg/dL), median (IQR)		142.5 (116.8; 197)	185 (136.8; 297.8)	0.054***
	Glycated hemoglobin (%), median (IQR)		7.1 (6.1; 8.4)	7.2 (6.3; 8.6)	0.700***
	Creatinine (mg/dL), median (IQR)		1.4 (0.9; 2.6)	1.3 (0.9; 2.3)	0.390***
	Urea (mg/dL), median (IQR)		66 (46; 133.8)	69 (55; 109)	0.853***
	Total cholesterol (mg/dL), median (IQR)		161.5 (122; 200.8)	165.5 (134.8; 185.5)	0.701***
	Low density lipoprotein (mg/dL), mean ± SD		93.2 ± 48.9	98.6 ± 33.7	0.618*
	High-density lipoprotein (mg/dL), mean ± SD		46.7 ± 14.9	41.3 ± 13.7	0.146*
	Triglycerides (mg/dL) , median (IQR)		112 (76; 187.5)	126 (98; 164.5)	0.383***
	Albuminuria, mg/g, median (IQR)		23.1 (8.1; 102.7)	53.4 (9.5; 126.9)	0.311***
Corticotherapy, n (%)			13 (43.3)	12 (40)	1.000**
	Daily prednisone dose (mg), median (IQR)		100 (90; 100)	80 (40; 100)	0.078***
	Duration, median (IQR)		7 (7; 8.5)	10 (7; 10)	0.114***

Primary and secondary outcomes are described in Table 2. CGM group had a significantly higher number of readings per day (six vs. four, p < 0.001), an improved time-in-range (100-180 mg/dL) (53.5% vs. 36.5%, p = 0.027), and less time above the target range (25.5% vs. 56.5%, p = 0.003), especially for values above 250 mg/dL (5% vs. 27.5%, p = 0.001). Although the CGM group had more readings below the target range (12 vs. 8, p = 0.047), there was no significant difference in the total number of hypoglycemia readings observed (one vs. zero, p = 0.107).

**Table 2:** Comparison of Outcomes CGM: continuous glucose monitoring; CBG: capillary blood glucose; IQR: interquartile range *Mann-Whitney U test; **Student’s t test for independent samples; ***Fisher exact test.

Result		CGM (n = 30)	CBG (n = 30)	p-value
Glycemic targets (%), median (IQR)				
	> 250 mg/dL	5 (0; 22.5)	27.5 (13; 39.5)	0.001*
	> 180 mg/dL	25.5 (9.3; 53.8)	56.5 (35; 71)	0.003*
	100-180 mg/dL	53.5 (34.3; 60.0)	35 (20.3; 48.5)	0.027*
	< 100 mg/dL	12 (5.3; 25.8)	8 (2.3; 11.8)	0.047*
	< 70 mg/dL	1 (0; 2.8)	0 (0; 1.8)	0.110*
Mean glucose (mg/dL)				
	Daily mean glucose, mean ± SD	161.9 ± 47.5	206.5 ± 47.7	<0.001**
	Fasting mean glucose, median (IQR)	116 (95; 143.8)	148.5 (115.5; 206.5)	0.002*
	Pre-prandial mean glucose, mean ± SD	168.4 ± 46.6	217.9 ± 49.9	<0.001**
	Post-prandial mean glucose, mean ± SD	166.4 ± 51.5	214.1 ± 58.1	0.001**
Glucose coefficient of variation (%), median (IQR)		34.2 (27.1; 39.2)	34.8 (32.1; 42.9)	0.181*
Hypoglycemia (n), median (IQR)				
	Total hypoglycemia	1 (0; 4.8)	0 (0; 2)	0.107*
	55–69 mg/dL	1 (0; 3.5)	0 (0; 2)	0.044*
	< 55 mg/dL	0 (0; 0,25)	0 (0; 0)	0,742*
	Nocturnal hypoglycemia	0 (0; 1)	0 (0; 0)	0.025*
Readings per day (n), median (IQR)		6 (5; 11.3)	4 (4; 5)	<0.001*
Length of stay (days), median (IQR)		22 (16; 32.8)	22 (16; 25.3)	0.455*
Inpatient mortality, n (%)		4 (13.3)	3 (10)	1.000***
Inpatient infection, n (%)		17 (56.7)	14 (46.7)	0.606***

In addition, the CGM group had a lower mean glucose level (161.9 mg/dL vs. 206.5 mg/dL, p < 0.001), with no significant difference in glucose coefficient of variation (34.2% vs. 34.8% p = 0.181). Moreover, the CGM group had significantly lower average fasting blood glucose (125.0 vs. 165.8; p = 0.014), pre-prandial (168.4 vs 217.9; p < 0.001), and postprandial (166.4 vs. 214.1; p = 0.001) values compared to the CBG group.

No differences were observed in length of hospital stay, reduction in mortality, or infection rate between the CGM and CBG groups (p = 0.455, p = 1.000, and p = 0.606, respectively).

## Discussion

The present study aimed to evaluate the impact of isCGM devices versus CBG on the glycemic control of inpatient type 2 diabetes on intensive insulin therapy.

Our results showed that the use of Freestyle Libre 2, an isCGM device, was associated with a significant improvement in time-in-range compared to CBG monitoring. Additionally, we found that patients using the CGM device had a significantly lower percentage of glucose readings above 180 mg/dL and 250 mg/dL. In the CGM group, we were able to achieve better glycemic control with fewer patients with hyperglycemia, but with no increased incidence of hypoglycemia. This outcome provides further evidence of the safety of these devices.

For inpatient management of hyperglycemia in noncritical care, the expert consensus recommends a target range of 100-180 mg/dL for noncritically ill patients with diabetes, as fasting glucose levels <100 mg/dL have been predictors of hypoglycemia and are associated with worse outcomes [13]. In our study, the CGM group spent a greater proportion of time above the target (12% vs. 8%, p = 0.047), mostly due to readings between 70 and 100 mg/dL, and without experiencing a higher occurrence of hypoglycemia. Although this may have been due to the monitoring protocol’s alarm setting and an overall low incidence of hypoglycemia in the study population, it also suggests the need for a possible revision of the glycemic target when using these devices.

The number of daily glucose readings in the CGM group closely approximated our hospital's recommended protocol of seven readings per day, indicating a superior adherence facilitated by these devices.

These findings suggest that the use of CGM devices may be a more effective tool for inpatient diabetes management than CBG monitoring, especially due to the increased adherence of the nursing team to this type of monitoring.

Although there were no significant effects on mortality, infection rate, or length of stay, it is important to acknowledge that the study was conducted during the COVID-19 pandemic and that 26.7% of the study population was admitted for COVID-19. This period was characterized by a mandatory minimum length of stay and an unusually high rate of mortality compared to pre-COVID-19 (6,62% inpatient mortality in 2021 vs. 3.17% in 2019, p < 0.001) which may have influenced the outcomes of the study.

As aforementioned, the American Diabetes Association 2023 diabetes guidelines recommend CBG as a glucose monitoring method for non-critically ill hospitalized patients with type 2 diabetes [1]. However, our study suggests that CGM may be a superior method for improving glycemic control, as supported by findings not yet described in the literature.

The only comparable and recent randomized clinical trial studied the application of rtCGM-guided insulin administration in 185 general medicine and surgery patients with type 1 and type 2 diabetes [14]. This study showed no difference in time-in-range (54.51% vs. 48.64%, p = 0.14) or in mean daily glucose (183.2 vs. 186.8 mg/dL, p = 0.36), contrary to our findings (time-in-range 53.5% vs. 36.5%, p = 0.027 and mean glucose 161.9 mg/dL vs. 206.5 mg/dL, p < 0.001). However, as with our study, no difference was observed in the reduction of hypoglycemia (36% vs. 39%; p=0.68), glucose coefficient of variation (27% vs. 26%; p = 0.33), length of stay (median eight vs. eight days, p = 0.79), or mortality, when compared to CBG.

Our study adds to the existing literature by reinforcing that the use of CGM devices can lead to improved glycemic control in hospitalized patients with type 2 diabetes on intensive insulin therapy.

Limitations of our study include being retrospective, being conducted in a single center, and having a relatively small sample size. Further multicenter prospective studies with larger sample sizes are needed to confirm our findings. Additionally, a blind CGM device was not used in the control group, and therefore the number of hypoglycemias in this group could possibly be underestimated. Also, the medical teams did not use the full potential of CGM, including the ambulatory glucose profile report, and inpatient management, which could have had an even bigger impact on the gap observed between groups. Finally, our study did not evaluate the cost-effectiveness of CGM devices compared to CBG monitoring, which is an important consideration for healthcare providers and is our future investigation goal.

## Conclusions

Our study suggests that the use of intermittently scanned CGM devices may be a more effective tool for inpatient diabetes management than CBG monitoring. The use of CGM devices was associated with improved glycemic control, including an increased time-in-range and reduced hyperglycemic events, being safe concerning hypoglycemias. Our findings support the use of CGM devices in the inpatient setting as a potential alternative to CBG monitoring. Further research is needed to evaluate the cost-effectiveness of CGM devices compared to CBG monitoring and to confirm our findings in larger, prospective, multicenter studies.
